# Misinformation about spinal manipulation and boosting immunity: an analysis of Twitter activity during the COVID-19 crisis

**DOI:** 10.1186/s12998-020-00319-4

**Published:** 2020-06-09

**Authors:** Greg Kawchuk, Jan Hartvigsen, Steen Harsted, Casper Glissmann Nim, Luana Nyirö

**Affiliations:** 1 Department of Physical Therapy, University of Alberta, Edmonton, Canada; 2 Department of Sports Science and Clinical Biomechanics, University of Southern Denmark, Odense, Denmark; 3 Nordic Institute of Chiropractic and Clinical Biomechanics, Odense, Denmark; 4 Medical Research Unit, Spinecentre of Southern Denmark, University Hospital of Southern Denmark, Middelfart, Denmark; 5 Department of Regional Health Research, University of Southern Denmark, Odense, Denmark; 6 Department of Chiropractic Medicine, Balgrist University Hospital, University of Zurich, Zürich, Switzerland

**Keywords:** Social media, Twitter, Spinal manipulation, Chiropractic, Misinformation, Immunity

## Abstract

**Background:**

Social media has become an increasingly important tool in monitoring the onset and spread of infectious diseases globally as well monitoring the spread of information about those diseases. This includes the spread of misinformation, which has been documented within the context of the emerging COVID-19 crisis. Understanding the creation, spread and uptake of social media misinformation is of critical importance to public safety. In this descriptive study, we detail Twitter activity regarding spinal manipulative therapy (SMT) and claims it increases, or “boosts”, immunity. Spinal manipulation is a common intervention used by many health professions, most commonly by chiropractors. There is no clinical evidence that SMT improves human immunity.

**Methods:**

Social media searching software (Talkwalker Quick Search) was used to describe Twitter activity regarding SMT and improving or boosting immunity. Searches were performed for the 3 months and 12 months before March 31, 2020 using terms related to 1) SMT, 2) the professions that most often provide SMT and 3) immunity. From these searches, we determined the magnitude and time course of Twitter activity then coded this activity into content that promoted or refuted a SMT/immunity link. Content themes, high-influence users and user demographics were then stratified as either promoting or refuting this linkage.

**Results:**

Twitter misinformation regarding a SMT/immunity link increased dramatically during the onset of the COVID crisis. Activity levels (number of tweets) and engagement scores (likes + retweets) were roughly equal between content promoting or refuting a SMT/immunity link, however, the potential reach (audience) of tweets refuting a SMT/immunity link was 3 times higher than those promoting a link. Users with the greatest influence on Twitter, as either promoters or refuters, were individuals, not institutions or organizations. The majority of tweets promoting a SMT/immunity link were generated in the USA while the majority of refuting tweets originated from Canada.

**Conclusion:**

Twitter activity about SMT and immunity increased during the COVID-19 crisis. Results from this work have the potential to help policy makers and others understand the impact of SMT misinformation and devise strategies to mitigate its impact.

## Introduction

More than half of all persons on earth (53.5%) are estimated to now have regular internet access with 47% in low-middle income countries and 86.6% in high income countries [1]. With this level of penetration, the internet is the most influential tool on earth for distributing information, whether it be accurate or otherwise. Therefore, understanding the creation, spread and uptake of internet misinformation is of critical importance [2] given that misinformation can be given credibility and create negative impacts [3, 4].

Social media has been used in recent decades to anticipate various health events including the spread of infectious disease [5] and new cases of back pain [6]. With recent advances in social media analytics, it is now possible to not only apply these tools to anticipate the onset and spread of various health conditions, but to also identify the onset and spread of information about those conditions. Specifically, various studies have been conducted that show how social media can be used in this regard [7], how social media is consumed [8] and how it can be used to set agendas [9, 10]. Importantly, social media is not always a positive force. Many publications now document how social media can create and disseminate misinformation [11–14]. Even in the short time since the COVID crisis was declared a pandemic on March 11, 2020 [15], several publications have now documented various types of misinformation arising during the COVID crisis [16–18] including potential treatments, methods of prevention and protection, dietary recommendations and disease transmission [19].

While all misinformation is concerning, the public does not expect misinformation to be propagated by regulated health professions whose activities are overseen for public protection. Unfortunately, this has not been the case during the COVID-19 outbreak. Claims that personal immunity can be improved or “boosted” through spinal manipulative therapy (Axén I Bergström C, Bronson M, Côté P, Glissman CN Goncalves G, Hebert J, Hertel AJ, Innes S, Larsen KO, Meyer A, Perle SM, O’Neill S, Weber K, Young K, Leboeuf-Yde C: Putting lives at risk: Misinformation, chiropractic and the COVID-19 pandemic, in submission), an intervention applied by many professions but most commonly by chiropractors [20], appeared on social media as the COVID crisis evolved. Not only is there no clinical evidence of this claim [21], major organizations representing those who provide SMT reacted immediately to condemn the promotion of this idea as potentially dangerous to public health [21–29].

In this descriptive study, we detail how Twitter activity can be used to not only document the magnitude and time course of misinformation describing a link between spinal manipulative therapy (SMT) and boosting immunity, but how social media activity promotes or refutes these claims. Specifically, our study aimed to answer the following research questions:

- Has Twitter activity describing a relation between SMT and “boosting” immunity increased during the COVID-19 crisis?
- What is the magnitude and engagement of Twitter activity that promotes or refutes an SMT/immunity link?
- Does Twitter activity differ between health professions that are mentioned in relation to SMT and immunity?
- What are the demographics (i.e. language, country) of Twitter authors who promote or refute a SMT/immunity link?

We anticipate that knowledge gained from answering these questions will be important not only in predicting future internet misinformation about SMT, but also in preventing and/or mitigating its impact.

## Methods

### Search

Social media searching was performed using Talkwalker Quick Search (Luxembourg, Luxembourg). Similar to tools used for searching health literature (e.g. EMBASE), Talkwalker performs searches of specific internet content including social media platforms, news agencies, forums and blogs. Talkwalker’s functionality allows searching to be limited to specific content sources, date ranges, electronic devices and many other parameters using standard Boolean syntax. Analysis of search results can be performed in several ways including descriptive metrics generated by Talkwalker using existing data (e.g. sex distribution), derived metrics generated by Talkwalker using artificial intelligence algorithms (e.g. sentiment) and user-generated metrics obtained by downloading raw search results directly into other software (e.g. Excel, SPSS).

For this project, Talkwalker searches were performed exclusively on Twitter for the 3 months before March 31, 2020. Twitter was searched preferentially for the following reasons. First, the entirety of Twitter is searchable (except for direct messaging which is a private discussion between Twitter users) compared to sources such as Facebook whose users must purposefully make their activity available for searching. Second, Twitter is a one-to-one communication model where direct dialogue is possible between all users compared to news media where unbalanced communication occurs through a one-to-many model. Finally, Twitter activity is unmoderated creating potential for a full range of conversation (except for content excluded by Twitter’s rules and policies).

Our primary search (Search #1) was constructed of three main components using Boolean syntax: 1) [procedure] terms related to SMT, 2) [profession] terms related to professions most often associated with SMT and 3) an immunity term [immun*]. In this study, we limited professions to be those that most often provide SMT (chiropractic, physiotherapy, naturopathy, osteopathy and naprapathy). No additional filters were used (e.g. language). Procedure terms included wildcard representations of words commonly used to describe SMT including manipulation, adjustment and SMT. Profession terms included wildcard card representations of chiropractic, physical therapy, naturopathy, osteopathy and naprapathy. As Talkwalker lacks the ability to perform Boolean operations *between* searches (i.e. union, intersection, difference), we performed additional searches to explore how search terms contributed to the primary search. Search #2 and Search #3 were performed to understand the impact of procedures and professions on the main search. Similarly, we conducted searches #4–8 to understand if procedure terms occurred more frequently for specific professions. Searches #9–13 were performed to understand how individual professions were linked specifically to immunity. Finally, Search #1 was performed again for the 12 months before March 31, 2020 as this is the longest period Talkwalker can search backwards in time (not listed in Table 1).

**Table 1:** Twitter searches performed in Talkwalker. Searches #1–13 were conducted over three months between January 01/01/2020 to March 31, 2020. Search #14 (not listed here) was a replicate of Search #1 conducted over the 12 months before March 31, 2020

#	Search components	Specific search terms
1	[procedures] OR [professions] AND [immun*]	(adjust* OR manipulat* OR smt OR chiro* OR physio* OR “physical therap*” OR naturo* OR osteo* OR napra*) AND immun*
2	[procedures] AND [immun*]	(adjust* OR manipulat* OR smt) AND immun*
3	[professions] AND [immun*]	(chiro* OR physio* OR “physical therap*” OR naturo* OR osteo* OR napra*) AND immun*
4	[procedures] OR [chiropractic] AND [immun*]	(adjust* OR manipulat* OR smt OR chiro*) AND immun*
5	[procedures] OR [physiotherapy] AND [immun*]	(adjust* OR manipulat* OR smt OR physio* OR “physical therap*”) AND immun*
6	[procedures] OR [naturopathy] AND [immun*]	(adjust* OR manipulat* OR smt OR naturo*) AND immun*
7	[procedures] OR [osteopathy] AND [immun*]	(adjust* OR manipulat* OR smt OR osteo*) AND immun*
8	[procedures] OR [naprapathy] AND [immun*]	(adjust* OR manipulat* OR smt OR napra*) AND immun*
9	[chiropractic] AND [immun*]	chiro* AND immun*
10	[physiotherapy] AND [immun*]	(physio* OR “physical therap*”) AND immun*
11	[naturopathy] AND [immun*]	naturo* AND immun*
12	[osteopathy] AND [immun*]	osteo* AND immun*
13	[naprapathy] AND [immun*]	napra* AND immun*

The above searches identified tweets that contained the search terms in the body of the tweet as words and/or hashtags (e.g. #chiropractic). For each individual tweet identified, multiple attributes describing its content were provided including date, creator, content, country of origin, language, likes, retweets, followers etc. A glossary of Twitter-related terms such as #hashtag can be found in Table 2.

**Table 2:** A glossary of Twitter-related terms

Engagement	The number of times a tweet is liked and retweeted.
Follower	A Twitter user who subscribes to the Tweets (i.e. posts) of another Twitter user.
Hashtag (#)	A word or phrase preceded by a hash sign (#) used on social media to identify a specific theme or topic.
Influencer	An individual who has the power to affect purchase decisions of others because of their authority, knowledge, position, or relationship with their audience (Talkwalker’s definition).
Like	When a Twitter user acknowledges another user’s tweet (i.e. post).
Mention	Any Twitter activity that contains the search terms (Tweets, retweets, likes etc.)
Potential Reach	The number of potential followers (i.e. subscribers) reached by the Tweet.
Retweet	When a tweet is retweeted (re-posted) by another Twitter user.
Sentiment Score	Sentiment is an expression of the emotional tone behind the tweet that attempts to summarize the attitudes and opinions being expressed. The sentiment score is an integer value which sums the sentiment values of individual mentions [30].
Tweet	A post on Twitter made by an individual on their own behalf or as a representative of a group/organization.

### Mentions over time

The above searches resulted in mentions (see Table 2) over time that were then tallied and plotted.

### Tone coding and sentiment

Tweets arising from Search #1 were first coded for their tone using the Twitter Tone Index (TTI). The TTI (Table 3) is a nominal index constructed for the purpose of this paper from a training set of 86 tweets that resulted in four coding options: 1) promoting a relation between SMT and/or a profession providing SMT and improved immunity, 2) refuting that same relation, 3) neutral content or 4) irrelevant content. This sample of 86 tweets was then scored independently by four evaluators (LN, SH, CN, JW) to calibrate their use of the TTI. This calibration resulted in 95% of tweets having at least three authors in agreement, and a Fleiss Kappa score of 0.85 interpreted as ‘almost perfect agreement’ [31]. These same evaluators then independently assessed each tweet arising from Search #1 using the TTI. Tweets not having at least 3 evaluators in agreement were discussed to agree on a majority TTI rating. Unresolved ties were broken by a fifth evaluator (GK). Additionally, the sentiment score of each tweet as determined by a proprietary Talkwalker artificial intelligence algorithm scored Tweets using positive or negative integers. The sentiment score is a rolling sum. If 3 Tweets have sentiment scores of 1, 2, 3 and another 3 Tweets have scores of − 1, − 2, − 3, then the resulting sentiment score for that topic is 0.

**Table 3:** Twitter Tone Index (TTI)

Tweet Content	Coding Description	Example Hit (bold = search terms)
Promoting	A tweet that suggests directly or indirectly that a SMT/profession improves or boosts immunity	-**#chiropractic** boosts your **immune** system up to 200%
Neutral	Factual, not misleading (as defined by WHO etc.)	-Wash your hands often. #**chiropractic** #**immunity**
Refuting	A tweet that directly or indirectly refutes a SMT/profession for promoting or boosting immunity.	**-Naturopathic** treatment can boost the **immune** system (screen capture). This is false!
Not Relevant	A tweet with unrelated content	**-**What are the roadblocks for treating **osteo**sarcoma with **immuno**therapy?

### Profession coding

Following TTI scoring, four evaluators (LN, SH, CN, JW) individually scored tweets arising from Search #1 regarding professions mentioned within each tweet (chiropractic, physical therapy, naturopathy, osteopathy, naprapathy). Tweets that did not mention a relevant profession were coded as “none mentioned”. Tweets not having at least 3 evaluators in agreement were discussed to agree on a majority rating. Unresolved ties were broken by a fifth evaluator (GK). Importantly, it was possible to code only whether tweets mentioned a profession; it was not possible to determine if or how the author was associated with a specific profession.

### Tweet themes (word frequency)

The content of all tweets obtained from Search #1 were pooled, analyzed for word frequency by a public website [32], then separated by TTI value (promoting or refuting).

### Influencers

Influencers were considered to be tweet authors having an engagement score (retweets + likes) of greater than zero. Tweets from each author were segregated by their TTI value and sorted by engagement score.

### Demographics

Descriptive statistics from Search #1 were derived for each Twitter user including language, and country of origin using geographical coordinates.

## Results

### Mentions over time

**Fig. 1:**
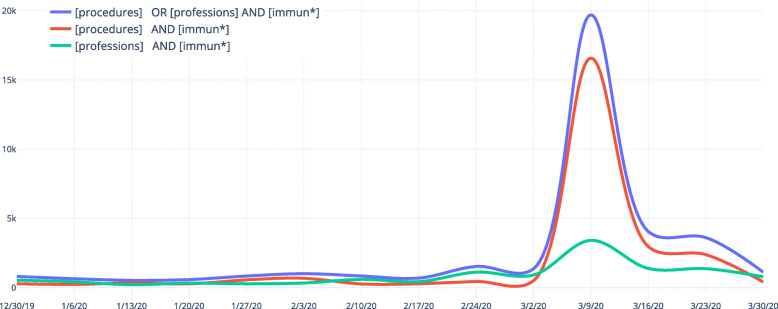
Total mentions for the 3 months before March 31, 2020 in total and segregated by procedure and profession (search #1–3 results). Procedures are terms related to SMT where health professions include chiropractic, physiotherapy, naturopathy, osteopathy and naprapthy

**Fig. 2:**
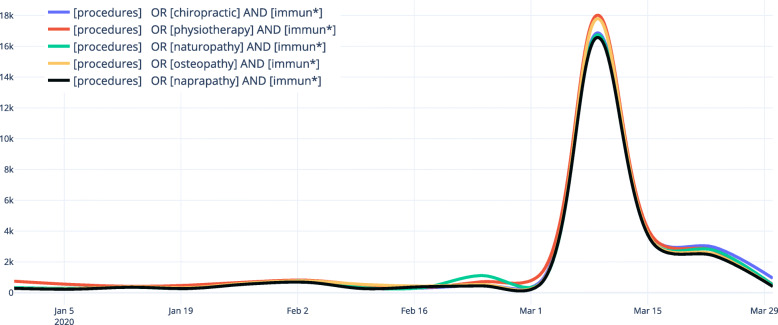
Total mentions for procedure terms in relation to each health professions and immunity for the 3 months before March 31, 2020 (search #4–8 results). Procedures are terms related to SMT where health professions include chiropractic, physiotherapy, naturopathy, osteopathy and naprapthy

**Fig. 3:**
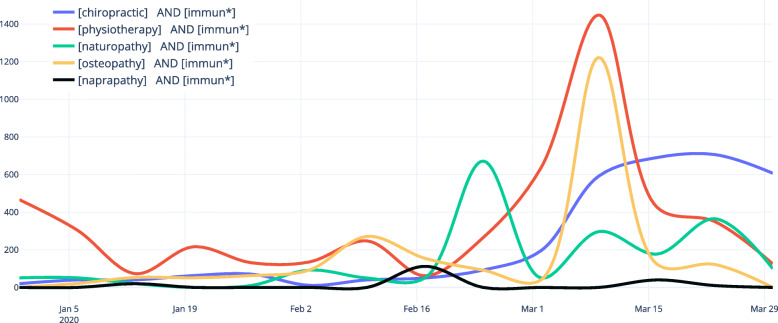
Total mentions for health professions in relation to immunity for the 3 months before March 31, 2020 (search #9–1 results). Procedures are terms related to SMT where health professions include chiropractic, physiotherapy, naturopathy, osteopathy and naprapthy

Total mentions over the 3 month study period are described in Table 4 and visualized in Figs. 1, 2 and 3. Graphing the results of search #1 displays the number of mentions over time. There is a peak of mentions on March 9th. (19.5 k mentions, Fig. 1). Searches 2 and 3 indicate that almost 26,000 of the mentions from Search #1 are procedure terms, while profession terms account for ~ 12,000 mentions in Search #1. Searches #4–8 demonstrate that search results varied between professions mentioned when all other terms were held constant. This finding, that Twitter activity is not distributed evenly between professions, was confirmed in Searches #9–13 (Fig. 3). This figure also shows that mentions involving a profession differ over time; Twitter activity related to most professions peaked near March 9, 2020 and then waned or oscillated. In contrast, Twitter activity related to mentions of “chiropractic” increased on March 9 and were sustained until the end of the study period.

**Table 4:** Mentions over time

#	Search	Mentions	Engagement	Sentiment	Potential Reach
1	(adjust* OR manipulat* OR smt OR chiro* OR physio* OR “physical therap*” OR naturo* OR osteo* OR napra*) AND immun*	37,308	98,699	+ 6%/− 24%	59,982,489
2	(adjust* OR manipulat* OR smt) AND immun*	26,159	65,335	+ 4%/− 29%	44,384,042
3	(chiro* OR physio* OR “physical therap*” OR naturo* OR osteo* OR napra*) AND immun*	12,105	34,323	+ 12%/−14%	16,478,381
4	(adjust* OR manipulat* OR smt OR chiro*) AND immun*	28,420	68,692	+ 5%/− 28%	51,023,438
5	(adjust* OR manipulat* OR smt OR physio* OR “physical therap*”) AND immun*	31,042	81,019	+ 5%/−26%	50,685,999
6	(adjust* OR manipulat* OR smt OR naturo*) AND immun*	27,983	68,884	+ 5%/−28%	45,634,407
7	(adjust* OR manipulat* OR smt OR osteo*) AND immun*	28,463	76,621	+ 4%/− 27%	46,086,149
8	(adjust* OR manipulat* OR smt OR napra*) AND immun*	26,341	65,701	+ 4%/−29%	44,405,522
9	chiro* AND immun*	3217	4316	+ 17%/− 25%	7,519,329
10	(physio* OR “physical therap*”) AND immun*	4893	15,693	+ 12%–9%	6,302,693
11	naturo* AND immun*	1986	3839	+ 11%/16%	1,441,794
12	osteo* AND immun*	2304	11,287	+ 6%/−4%	1,702,107
13	napra* AND immun*	0	0	N/A	0

**Fig. 4:**
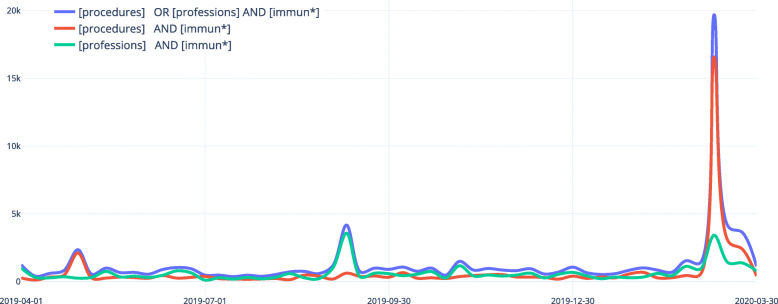
Baseline Twitter activity over the 12 months before March 31, 2020 (search #14 results). Procedures are terms related to SMT where health professions include chiropractic, physiotherapy, naturopathy, osteopathy and naprapthy

In the 12 months before March 31, 2020 (Fig. 4), baseline Twitter activity consisted of a relatively low volume of mentions punctuated by small activity peaks. This baseline activity preceded a large activity peak coinciding with the onset of the COVID crisis.

**Fig. 5:**
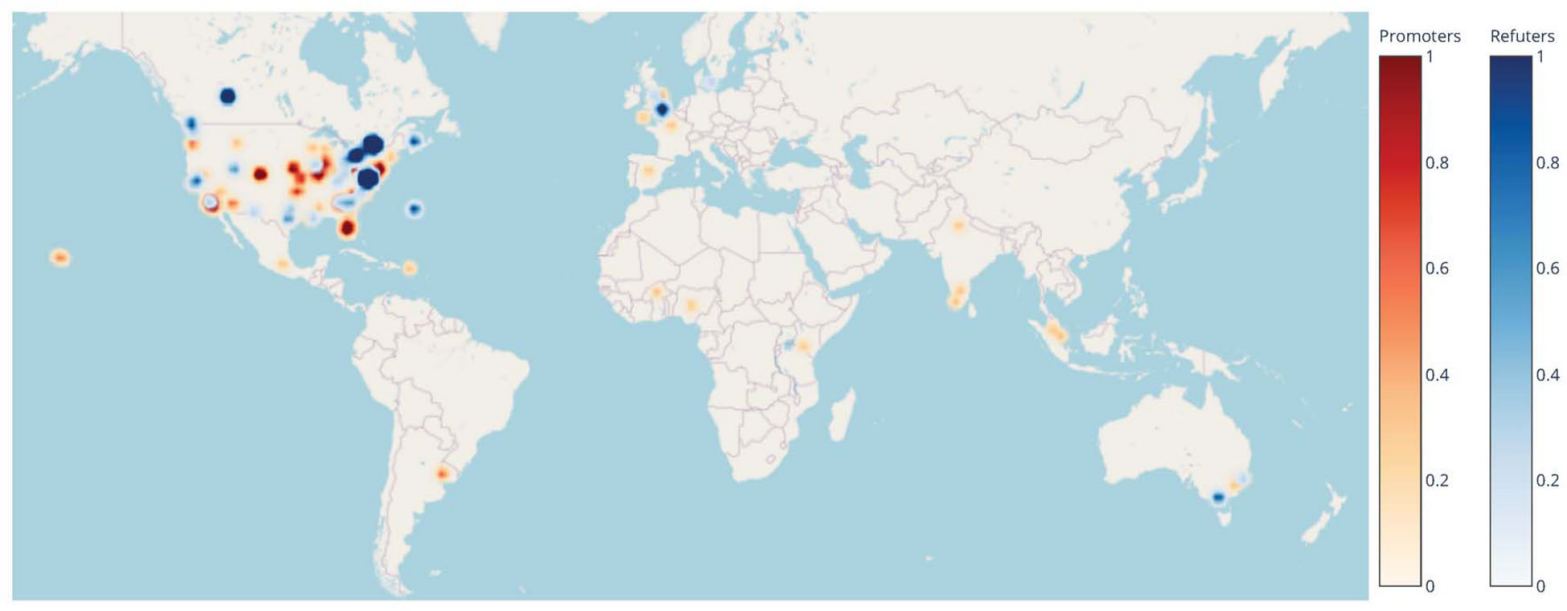
Global heat map of tweet location stratified by those promoting or refuting a message of boosting immunity

### Tweet coding and sentiment

There were 1118 individual tweets generated from Search #1 (Table 5). When coded to the TTI, 778 tweets were classified as not relevant with the remaining tweets divided between promoting (187 (24%)), refuting (141 (18%)) and neutral (12 (2%)). Although both promoting and refuting tweets were similar in their engagement scores (3319 vs. 3590), refuting tweets had a potential reach that was 3 times greater than promoting tweets (4,626,820 vs. 1,558,937). Overall, Talkwalker sentiment scores were positive for promoting tweets and negative for refuting tweets.

**Fig. 6:**
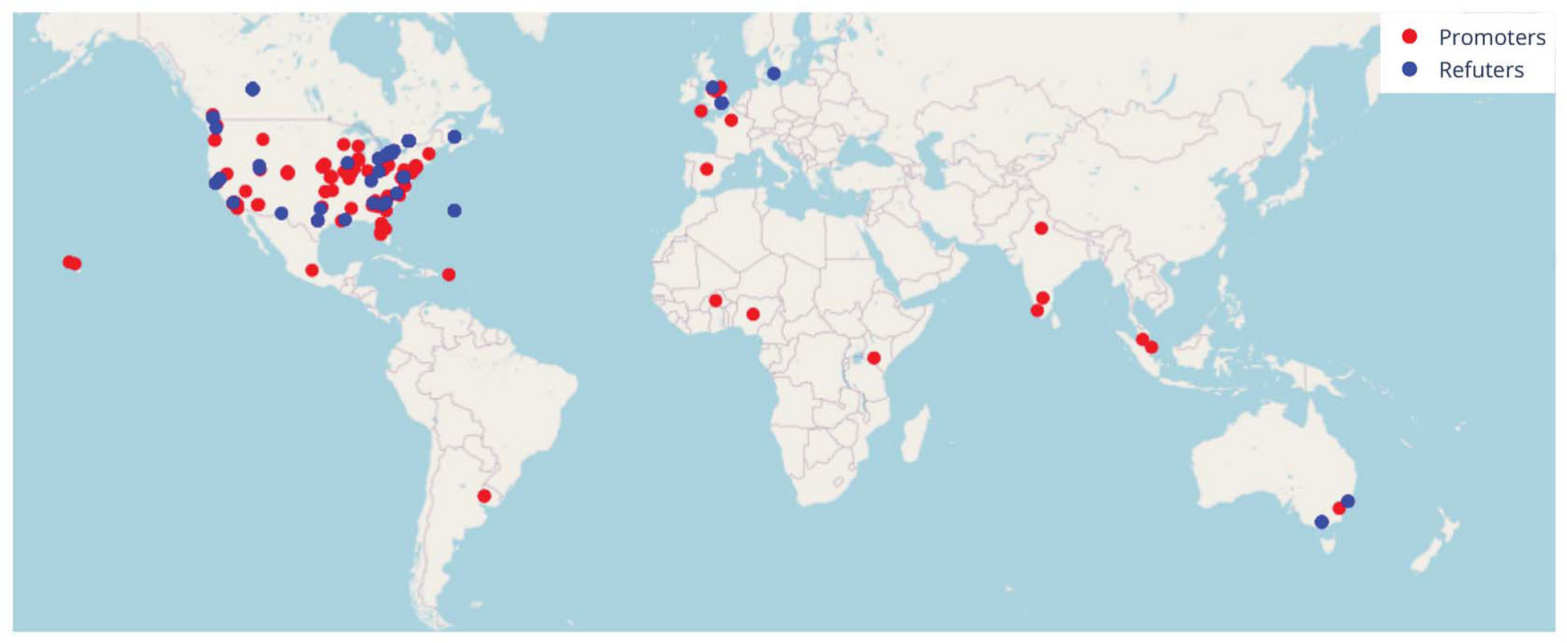
Global tweet location stratified by those promoting or refuting a message of boosting immunity

**Table 5:** Tweet coding for tone, mentioned profession and sentiment

Tone	Profession mentioned	Count	Reach	Engagement	Retweets	Likes	Sentiment
Neutral	Chiropractic	3	12,807	4	2	2	0
	Naturopathy	3	16,522	32	7	25	0
	None Mentioned	2	6126	11	10	1	5
	Osteopathy+Physiotherapy+Other Profession	1	513	0	0	0	5
	Physiotherapy	3	3504	22	1	21	0
Neutral Total		12	39,472	69	20	49	10
Not relevant	Chiropractic	2	305	3	1	2	−5
	Chiropractic+Other Profession	1	1664	8	0	8	5
	Naturopathy	1	1624	4	0	4	−5
	None Mentioned	774	22,822,193	74,621	24,049	50,572	− 720
Not relevant Total		778	22,825,786	74,636	24,050	50,586	− 725
Promoting	Chiropractic	108	40,341	163	44	119	210
	Naturopathy	38	201,344	185	44	141	70
	Naturopathy+Other Profession	2	53,525	1712	511	1201	5
	None Mentioned	30	1,261,529	1242	388	854	20
	Osteopathy	2	646	1	1	0	0
	Other Profession	4	989	11	3	8	10
	Physiotherapy	3	563	5	2	3	−5
Promoting Total		187	1,558,937	3319	993	2326	310
Refuting	Chiropractic	117	4,263,261	2393	557	1836	− 210
	Chiropractic+Naturopathy	4	123,829	670	209	461	−5
	Chiropractic+Naturopathy+Other Profession	2	107,435	274	87	187	−5
	Naturopathy	14	130,086	237	64	173	−30
	None Mentioned	3	1755	5	0	5	−10
	Physiotherapy+Other Profession	1	454	11	1	10	−5
Refuting Total		141	4,626,820	3590	918	2672	− 265
**Grand Total**		**1118**	**29,051,015**	**81,614**	**25,981**	**55,633**	**− 670**

When these 1118 tweets were coded for the 5 professions related to SMT, there were 809 tweets where a profession was not mentioned and 7 tweets mentioning an irrelevant profession. Of tweets mentioning a profession relevant to SMT, some mentioned a single profession while others mentioned multiple professions; a distinction retained in our coding (Table 5). From all mentions of professions (11280), chiropractic was mentioned most often (237 (21%)) compared to naturopathy (64 (6%)). Tweets mentioning chiropractic had a potential reach of 4,549,642 Twitter users with a total engagement of 3515 and a total sentiment score of − 10 while for naturopathy, the potential reach was 634,365 with a total engagement of 3114 and a total sentiment score of + 30.

When analyzing mentions of profession for tweets that either promoted (189 mentions) or refuted (148 mentions) a link between SMT and immunity, chiropractic was mentioned 108/189 times (57%) in promoting tweets and 123/148 times (83%) in refuting tweets. Naturopathy was the next-most mentioned profession with 40/189 (21%) mentions in promoting tweets and 20/148 (14%) mentions in refuting tweets.

### Tweet themes (word frequency)

The major themes (frequent words) contained within the 1118 tweets from Search #1 are presented in Table 6. Terms related to chiropractic and the term “boost” were the most common themes with “evidence” mentioned only in the refuting themes. The expression “adjustment” was used more frequently than the expression “manipulation” or “spinal manipulation”.

**Table 6:** Top 20 themes (word frequency) contained in Tweets for all tweets and those scored as promoting or refuting misinformation

Rank	Promoting	Occurrences		Refuting	Occurrences
1	chiropractic	81		boost	78
2	boost	44		chiropractors	50
3	help	40		chiropractic	50
4	immunity	36		chiropractor	36
5	care	35		adjustments	31
6	#chiropractic	30		prevent	27
7	health	26		covid	27
8	healthy	23		immunity	24
9	adjustments	23		cbc	24
10	body	21		evidence	24
11	virus	20		#coronavirus	22
12	#coronavirus	19		pandemic	22
13	coronavirus	18		claiming	20
14	vitamin	16		#covid19	19
15	#immunesystem	15		posts	18
16	systems	14		claims	16
17	adjustment	14		ontario	15
18	sleep	13		spinal	15
19	#health	12		people	15
20	naturopath	12		help	14

### Influencers

In total, there were 132 Twitter authors having engagement scores of > 0 for the study period. Table 7 stratifies these authors into those creating promoting or refuting tweets. While total engagement was similar between both these groups, the potential reach in the refuting group was 3.29 times larger.

**Table 7:** Tweets of the top 25 promoting and refuting influencers (of 132) sorted by descending engagement scores where engagement scores were > 0. Row values of SumReach and SumEngagement are the total potential reach and engagement respectively for all posts by that author. Totals at the bottom of the table are for all 132 authors having an engagement score of > 0 (row data for authors ranked 26–66 are not shown)

Promoting (25 of 66)				Refuting (25 of 66)			
Rank	Count	SumReach	SumEngagement	Rank	Count	SumReach	SumEngagement
1	1	53,525	1712	1	9	483,032	1422
2	1	3099	741	2	1	16,078	369
3	1	31,796	165	3	2	93,324	276
4	1	1,133,212	141	4	30	88,888	257
5	1	1642	99	5	1	211	157
6	1	48,991	98	6	1	19,188	139
7	1	464	84	7	3	11,780	118
8	1	18,230	28	8	1	2,878,804	73
9	1	3714	23	9	1	8731	69
10	1	77	19	10	1	188,258	59
11	1	3390	17	11	1	16,910	58
12	1	727	17	12	1	11,531	49
13	1	7487	11	13	1	7334	49
14	1	127	11	14	1	847	46
15	1	913	10	15	1	3106	45
16	1	721	8	16	18	3904	32
17	1	7380	7	17	1	24,573	31
18	1	2773	7	18	3	9745	30
19	1	2074	7	19	2	4110	25
20	1	775	7	20	1	2175	24
21	1	68	6	21	1	6710	21
22	1	34	6	22	1	4100	15
23	1	3	6	23	3	3994	15
24	1	346	5	24	1	17,692	14
25	1	317	5	25	1	924	13

### Demographics

Demographics from Search # 1 were segregated by TTI value (promoting or refuting) and are displayed in Table 8. For both promoting and refuting tweets, the majority of authors were male. English was the predominant language. The country of origin differed between promoting and refuting tweets. Tweets promoting a link between spinal manipulation and immunity were created most often in the United States. Canada generated the greatest number tweets refuting this link (Table 8). Figures 5 and 6 were plotted using longitude and latitude data associated with each tweet.

**Table 8:** Demographics describing sex, age, and language of Twitter content related to all searches

**Sex**	**Promoting Authors**	**Refuting Authors**
Female	51	19
Male	78	98
Unknown	58	24
**Language**	**Promoting Authors**	**Refuting Authors**
English	186	141
French	1	0
**Country**	**Promoting Tweets**	**Refuting Tweets**
United States	89,249	28,278
Canada	3167	38,488
United Kingdom	2586	3664
Australia	1351	1989
Uruguay	1259	0
Puerto Rico	1070	0
Mexico	979	0
France	925	0
Kenya	898	0
Spain	643	0
Malaysia	423	0
India	413	0
Nigeria	225	0
Singapore	155	0
Burkina Faso	54	0
Burmuda	0	2356
Denmark	0	667

## Discussion

This paper presents the novel finding that Twitter misinformation regarding a SMT/immunity link increased dramatically during the onset of the COVID crisis. Further, activity levels and engagement were roughly equal between tweets promoting a SMT/immunity link and tweets refuting this claim. Interestingly, the potential audience (reach) of tweets refuting these claims was 3 times higher than those promoting these claims.

### Mentions over time

The majority of search results (i.e. mentions) from Search #1 were coded as not relevant on the TTI and did not mention a specific profession (778 (70%)). Combined with tweets having a neutral tone (12 (2%)), the vast majority of mentions from Search #1 were not relevant to our analysis. While our search terms could have been made more restrictive to reduce this number of irrelevant mentions (e.g. using “spinal manip*”), we preferred to err on the side of having too many search results that were then coded by our team rather than construct too narrow a search that potentially missed relevant tweets.

Clearly, Twitter mentions about a SMT/immunity link increased during the onset of the COVID-19 crisis with peak activity being almost 5 x higher on March 9, 2020 (19.7 k mentions) compared to any other peak activity in the prior 12 months (e.g. September 9, 2019, 4.2 k mentions). This suggests that mentions during the COVID-19 crisis were intentional and not an aberration of baseline activity. To further assess baseline Twitter activity, we evaluated the second largest peak of mentions in the preceding 12 months (September 9, 2019, 4.2 k mentions). This activity consisted almost entirely of twitter content unrelated to the aims of the paper. However, our analysis did reveal a smaller activity peak on October 21, 2019 that appeared to be related to an automated message delivered from a web content subscription service.

*“Chiropractic care can improve your immune system, mobility, strength, and so much more. If you want to see a positive change in your health, schedule an appointment with us”.*

This specific tweet appeared in 17/21 unique tweets on October 21, 2019 within hours of each other. These 17 tweets generated a total potential reach of 54 users and an engagement score of 1 (retweets + likes). In contrast, a single tweet in the same time period that refuted this message generated a potential reach of 2657 users with an engagement score of 25.

### Tweet coding and sentiment

When a tweet is made, it automatically goes out to all persons who follow (i.e. subscribe) the author’s Twitter account. While sometimes the potential reach of that author is in the thousands or even millions, there is no guarantee that their followers open their device and see the tweet let alone read it. Therefore, the number of followers, or the potential reach of an author is a measure of the *potential* impact of a tweet. In contrast, if someone acknowledges a tweet by giving it a like or retweeting it (i.e. rebroadcasting it to their own followers), this confirms that the original tweet was both read and acknowledged indicating a true interaction between users. Considering this, tweets that refute a SMT/immunity link had almost 3 times the potential reach compared to those that promoted this link although the engagement between these two groups was similar. This is an important finding as it suggests that promoting tweets create as much engagement as refuting tweets but with the important note that refuting tweets have the potential of reaching many more persons with their message. Still, it is highly likely that the engagement and potential reach of promoting and refuting tweets have differing audiences who are unaligned in their belief systems about SMT and immunity [33].

Regarding professions mentioned in tweets, our coding revealed that our initial wildcard search terms for physiotherapy and osteopathy were too broad resulting in tweets having topics related to physiology and osteology for example. Following coding to eliminate these tweets, chiropractic was the profession most often referenced with 4 times more mentions than the next profession (naturopathy). These data suggest that the majority of twitter activity regarding a SMT/immunity link is associated with the chiropractic profession with the total number of posts being roughly equal between those promoting and those refuting this link.

### Tweets themes

Tweet themes do not appear to be a good indicator of the impact of specific content as the frequency of the theme is not related to the potential reach or engagement associated with the message; an infrequent theme may be posted in a tweet with far greater reach and engagement than higher ranked themes with lower reach and engagement.

### Influencers

The top influencers for tweets promoting and refuting a SMT/immunity link each had engagement scores that were ~ 1000 points higher than the next influencer. This shows influence distribution is not equal within each group. Even more so, top influencers appear to be individuals and not academic institutions, regulatory bodies or professional organizations. Thus, few institutions (e.g. universities, associations) were identified as influencers although some individuals with a specific institutional affiliation could be identified. Although Twitter data is publicly available, and Twitter users agree to make their information available publicly, we have chosen not to identify user names of influencers so as not to inadvertently legitimize those who promote misinformation.

### Demographics and global distribution

The majority of those promoting or refuting a SMT/immunity link were male and English speakers. Interestingly, tweets promoting a SMT/immunity link most commonly originated in the United States. Although tweets rarely were affiliated with specific institutions, we note that the majority of chiropractic, naturopathic and osteopathic schools in the world are in the United States. In contrast, the majority of tweets refuting a SMT/immunity link were from Canada which suggests that geographic proximity between countries is not a factor in establishing a position on this topic. These data likely reflect the distribution of Twitter use around the world. The United States is the number one user of Twitter with Japan in second place and Canada in 12th place [34].

### Strengths and limitations

Results from this work have the potential to help policy makers and others understand the impact of SMT misinformation and devise strategies to mitigate its impact. Specifically, our results suggest that while the potential reach of messaging that refutes misinformation about SMT was substantial, very few institutions added to this total. Assuming that most institutions related to SMT stand to gain from combating misinformation about SMT (educational programs, associations, regulators, health care administrators etc), these same institutions should re-evaluate their social media strategies lest their silence be taken to be complicit of misinformation or lead to their own demise from an erosion of public trust.

The results reported here are different from those presented previously by investigators who explored chiropractic messaging on Twitter in December of 2015 [35]. In this prior work, Tweets refuting claims about questionable benefits from SMT, including changes in immunity, appeared to be less in proportion compared to those promoting such claims. Possible explanations for these incongruent results include the methodologies used, the year/month of data collection and an increasing awareness of social media misinformation especially during the covid crisis.

While Talkwalker can assess other electronic data sources, only Twitter provides full access to its “firehose”, the entirety of its activity except for direct messaging between users (a private channel of communication between users). As a result, the data from this paper are presumed to be robust in that they represent all activity taking place on a single social media platform although search results from Talkwalker have not been compared against other services/techniques for accessing Twitter data.

Although Twitter provides a window into conversations within a social media community, it is limited in that it does not represent all persons in the world. Presently, Twitter ranks 13th in total monthly users; Facebook has 2.45 billion active monthly users compared to Twitter’s 340 million [36].

Some of the data used in this study were obtained from proprietary algorithms available from TalkerWalker Quick Search but whose methods of calculation were not available to us (e.g. sentiment scores). Similarly, Talkwalker Quick Search uses artificial intelligence to derive some demographic information not directly included in Twitter user profiles (age, occupation and interests). These proprietary metrics of defining user profiles were not used in our analysis.

## Conclusion

Twitter activity regarding misinformation about spinal manipulation and immunity increased above baseline levels during the COVID crisis. Direct Twitter activity (posts, likes, retweets, engagement) was similar between tweets promoting and refuting a SMT/immunity link. Importantly, tweets refuting a SMT/immunity link had the potential to be viewed by 3 times more people than tweets promoting this link. Whether promoting or refuting in tone, the chiropractic profession was most often mentioned in tweets compared to other professions associated with SMT provision. Results from this work have the potential to help policy makers and others understand the impact of SMT misinformation and devise strategies to mitigate its impact.

## Data Availability

All data generated or analysed during this study are included in this published article.

## Abbreviations

COVID: Coronavirus Disease
SMT: Spinal Manipulative Therapy
TTI: Twitter Tone Index
